# Histone Deacetylase 1 and 3 Regulate the Mesodermal Lineage Commitment of Mouse Embryonic Stem Cells

**DOI:** 10.1371/journal.pone.0113262

**Published:** 2014-11-20

**Authors:** Weiying Lv, Xudong Guo, Guiying Wang, Yanxin Xu, Jiuhong Kang

**Affiliations:** Clinical and Translational Research Center of Shanghai First Maternity and Infant Health Hospital, Shanghai Key Laboratory of Signaling and Disease Research, School of Life Science and Technology, Tongji University, Shanghai, P.R. China; Michigan State University, United States of America

## Abstract

The important role of histone acetylation alteration has become increasingly recognized in mesodermal lineage differentiation and development. However, the contribution of individual histone deacetylases (HDACs) to mesoderm specification remains poorly understood. In this report, we found that trichostatin A (TSA), an inhibitor of histone deacetylase (HDACi), could induce early differentiation of embryonic stem cells (ESCs) and promote mesodermal lineage differentiation. Further analysis showed that the expression levels of HDAC1 and 3 are decreased gradually during ESCs differentiation. Ectopic expression of HDAC1 or 3 significantly inhibited differentiation into the mesodermal lineage. By contrast, loss of either HDAC1 or 3 enhanced the mesodermal differentiation of ESCs. Additionally, we demonstrated that the activity of HDAC1 and 3 is indeed required for the regulation of mesoderm gene expression. Furthermore, HDAC1 and 3 were found to interact physically with the T-box transcription factor T/Bry, which is critical for mesodermal lineage commitment. These findings indicate a key mechanism for the specific role of HDAC1 and 3 in mammalian mesoderm specification.

## Introduction

Embryonic stem cells (ESCs) are derived from inner cell mass (ICM) and are distinguished from other cell types by their unique properties to maintain self-renewal and differentiate into multiple lineages [1]. These processes are controlled by extrinsic and intrinsic molecules that affect signal transduction, transcription regulation and epigenetic modification. Lineage-specific transcription factors have proved to be the dominant factors in the precise and sequential regulation of germ-layer differentiation [2]. Additionally, the accessibility of genomic DNA to transcription factors depends on dynamic changes in local chromatin architecture. Epigenetic mechanisms, especially histone acetylation, have recently become important in the research of stem cell differentiation and individual development in mammals [3]–[5]. Histone acetyltransferases (HATs) and histone deacetylases (HDACs) are responsible for relaxing (increasing gene expression) or condensing (inhibiting gene transcription) chromatin structure, respectively [6]. The cooperation of transcription factors with HATs and HDACs establishes and maintains specific patterns of gene expression in the multiple processes of ESCs and plays a key role in lineage specification and mammalian development.

The main function of HDACs is to remove acetyl groups from the N-acetyl lysines on histones, thus modifying chromatin structure and gene transcription [7]. The HDAC family contains 18 enzymes that are grouped into four classes: class I (HDAC1, 2, 3, and 8), class II (HDAC4, 5, 6, 7, 9, and 10), and class IV (HDAC11), which are called classical HDACs, and class III (SIRT1-7) [8]. Class I HDAC proteins are widely expressed and are mainly present in the nucleus, where they mostly modulate gene transcription [9]. The wide expression of class I HDACs suggests key roles for their activity in development. Knockout phenotypes of class I HDACs in mice have showed that they are involved in cell proliferation and differentiation [10]. Deletion of HDAC1 in mice results in embryonic lethality around embryonic day E10.5 [11],[12]. Although HDAC1 and HDAC2 exhibit a high degree of similarity (85%) [13], mice lacking HDAC2 successfully undergo the embryogenesis phase and survive until the perinatal period [14],[15]. Disruption of HDAC3 also results in embryonic lethality around E9.5 owing to gastrulation defects [16]. The knockout phenotype of HDAC8 remains undetermined [17]. Obviously, the above-mentioned studies suggest crucial roles of class I HDACs in the well-organized embryonic development. However, the specific and distinct roles of each member of class I HDACs in cell differentiation and development remain uncharacterized.

The activities of HDACs are precisely regulated by multiple mechanisms, including post-translational modification, subcellular localization, and protein-protein interaction. HDACs mostly interact together with several complexes, such as Sin3A, NuRD, CoREST, and NODE in mammalian cells [18]–[20]. The HDAC/Sin3A complex could modulate the transcriptional repressor activity of Nkx3.2 and Nkx2.2 via interacting with HDAC1 [21]. HDAC also inhibits the transcriptional activity of Nkx2.5 and other transcriptional factors (GATA2, RUNX2, and MEF2) via direct interaction, impairing cardiac development [22]. The T-box transcription factor T/Bry, which is evolutionarily conserved, is a well-known intrinsic molecule that is required for the proper specification of the mesodermal lineage [23]. Additionally, T^-/-^ embryos show deficiency of the posterior mesoderm' and impair the development of the primitive streak (PS), leading to embryonic lethality at approximately E10.5 [24]. However, whether HDACs have any roles in T-involved mesoderm specification remains to be clearly defined.

In the present study, we used a HDAC inhibitor (trichostatin A; TSA) to examine the function and regulation of class I HDACs during the early differentiation of stem cells. We also demonstrated that HDAC1 and 3 (but not HDAC2 or 8) are gradually decreased during differentiation and significantly inhibit the differentiation of ESCs into the mesodermal lineage. Furthermore, we demonstrated that HDAC1 and 3 physically interact with the T-box transcription factor T/Bry to repress mesodermal lineage commitment.

## Results

### TSA induces early differentiation of ESCs and promotes mesodermal lineage differentiation

**Figure 1:**
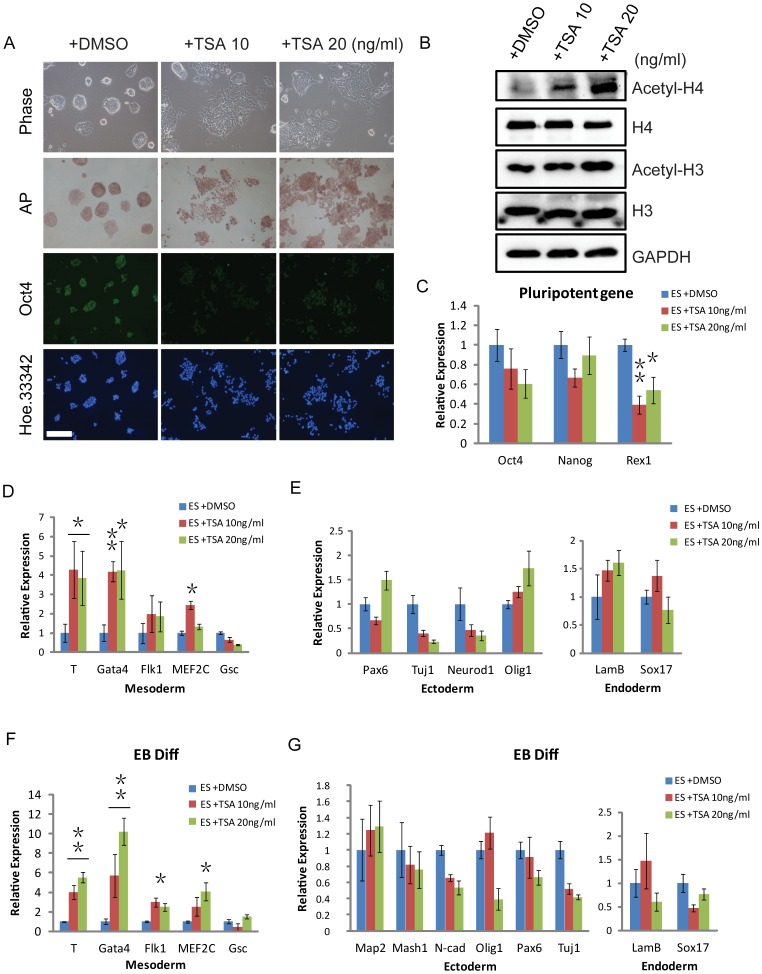
TSA induces early differentiation of ESCs and promotes mesodermal lineage differentiation. (*A*) Bright-field images, alkaline phosphatase staining of ESCs and representative immunofluorescence images of Oct4 staining in control or TSA-treated ESCs (10 and 20 ng/ml) in the presence of LIF. (*B*) Western blotting verification of H3, acetyl-H3, H4, and acetyl-H4 in control or TSA-treated ESCs (10 and 20 ng/ml). GAPDH was used as a loading control. (*C*) The relative expression levels of Oct4, Nanog, and Rex1 mRNA in control or TSA-treated ESCs (10 and 20 ng/ml). (*D, E*) QRT-PCR analysis for marker genes of three germ layers (endoderm, mesoderm and ectoderm) in control or TSA-treated ESCs (10 and 20 ng/ml), under the monolayer differentiation condition without LIF. The cells were treated by TSA after removing LIF for 24h and collected mRNA for QRT-PCR analysis at day 3 of monolayer differentiation. (*F, G*) QRT-PCR analysis for marker genes of the three germ layers in control or TSA-treated ESCs (10 and 20 ng/ml) during EB differentiation. The EBs was treated by TSA from day 2 to 6 of EB differentiation. Data are expressed as means ± SD. Statistical significance was assessed by two-tailed Student's t test. ***, P<0.001; **, P<0.01; *, P<0.05.

Treatment of aggregated P19 cells with the histone deacetylase inhibitor TSA could induce the entry of mesodermal cells into the cardiac muscle lineage [5]. We therefore used TSA to examine the role of histone acetylation in the early differentiation of ESCs. We first tested the effect of different TSA concentrations (10 and 20 ng/ml) on cultivated ESCs in the presence of LIF. We observed morphological changes following TSA treatment (Figure 1A). Figure 1A showed phase contrast morphology and alkaline phosphatase staining (AP) of ESCs treated with 10 and 20 ng/ml TSA for 24 h. Western blotting showed the protein levels of acetyl-H4 and acetyl-H3 were significantly increased with TSA treatment (10 and 20 ng/ml) (Figure 1B). After TSA treatment, the colonies mostly became separated and flattened, containing a mixed population of AP-positive and -negative cells (Figure 1A). The TSA-treated cells showed a loose morphology and a reduced Oct4 level (Figure 1A). The mRNA level of the pluripotent markers Oct4 and Nanog, particularly for Rex1, was markedly decreased in the ESCs treated by TSA for 24 h (Figure 1C). Next, we performed QRT-PCR to detect the marker genes of three germ layers (endoderm, mesoderm and ectoderm) in control or TSA-treated ESCs (10 and 20 ng/ml) in both the monolayer differentiation condition without LIF (Figure 1D-E) and the embryoid body (EB) differentiation condition (Figure 1F–G). TSA treatment significantly increased the important marker genes of mesodermal differentiation under the two differentiation conditions (Figure 1D and 1F). Taken together, our finding suggested that TSA could disrupt the undifferentiated state, induce the early differentiation of ESCs and prefer differentiation toward the mesodermal lineage.

### The expression levels of HDAC1 and 3 are decreased during differentiation

**Figure 2:**
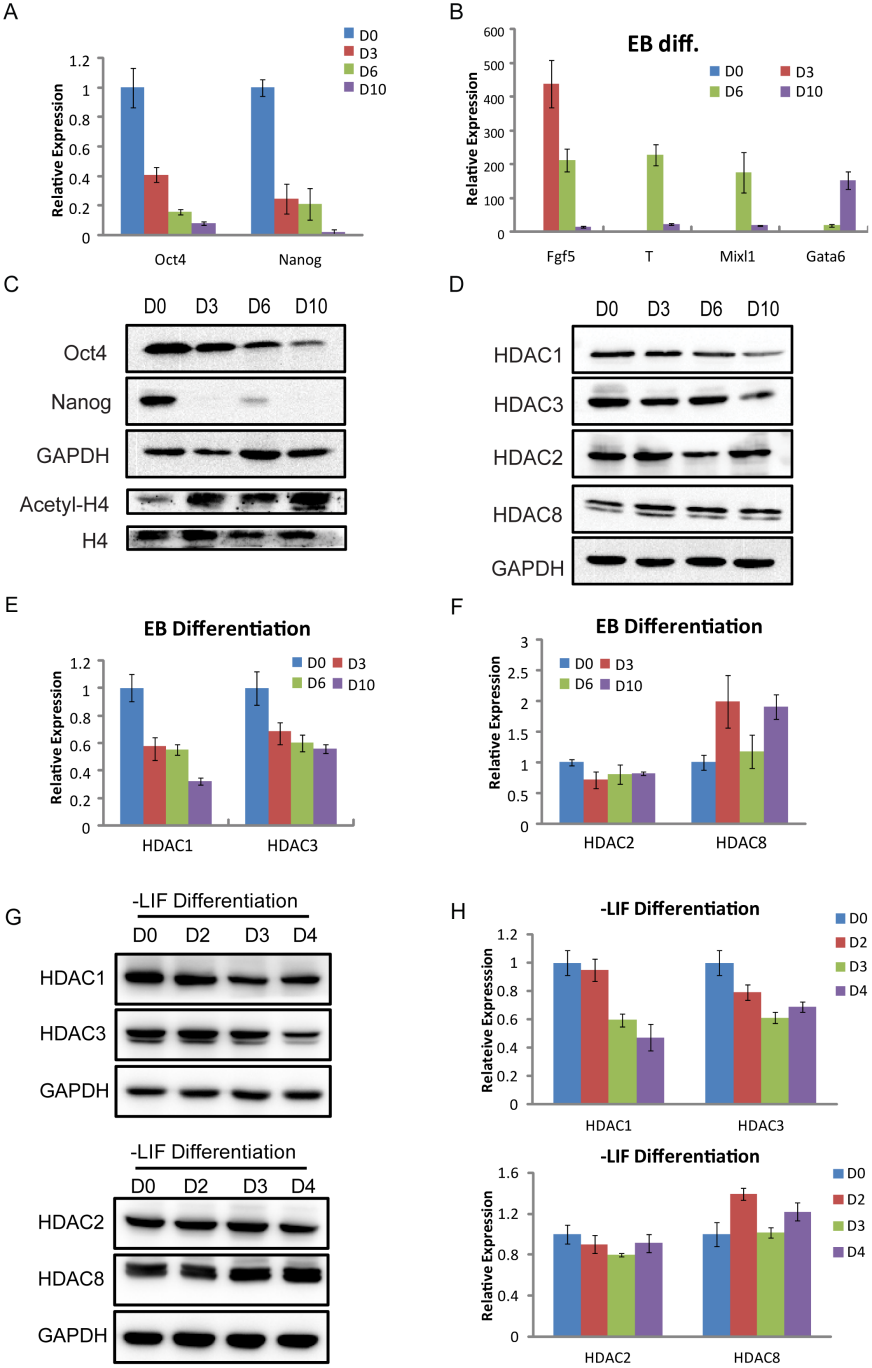
The expression levels of HDAC1 and 3 are decreased during differentiation. (*A*) QRT-PCR for genes characteristic of undifferentiated stem cells (Oct4, Nanog) was performed as indicated on mRNA collected at days 0, 3, 6, and 10 during EB differentiation. (*B*) The relative expression levels of marker genes for three germ layers (endoderm, Gata6; mesoderm, T, Mixl1; primitive ectoderm, Fgf5) at days 0, 3, 6, and 10 during EB differentiation. (*C*) Western blotting verification for genes characteristic of undifferentiated stem cells (Oct4, Nanog) was performed as indicated on protein samples collected at days 0, 3, 6, and 10 during EB differentiation. The expression level of global acetyl-H4 was increasing during EB differentiation. GAPDH and H4 were used as loading controls. (*D*) Western blotting verification for class I HDAC members (HDAC1, 2, 3, and 8) at the indicated days 0, 3, 6, and 10 during EB differentiation. GAPDH was used as a loading control. (*E, F*) QRT-PCR analysis for the expression levels of class I HDAC members (HDAC1, 2, 3, and 8) at the indicated days 0, 3, 6, and 10 during EB differentiation. (*G, H*) Western blotting and QRT-PCR analysis for the expression levels of HDAC members (HDAC1, 2, 3, and 8) at days 0, 2, 3, and 4 during differentiation without LIF.

As a first step in investigating the mechanism of class I HDAC members in the regulation of gene expression, we analyzed whether the expression levels of four class I HDAC members are altered during EB differentiation. First, analysis of mRNA extracted from EBs at days 0, 3, 6, and 10 showed a significant decrease in Oct4 and Nanog expression with time (Figure 2A), a finding that is consistent with the protein levels of Oct4 and Nanog decreasing during differentiation (Figure 2C), indicating that our strategy of EB differentiation was appropriate for subsequent studies. Moreover, we detected the mRNA levels of marker genes for the three germ layers (endoderm, Gata6; mesoderm, T, Mixl1; primitive ectoderm, Fgf5) at the indicated days 0, 3, 6, and 10 of EB differentiation to further confirm the differentiation protocol (Figure 2B). Interestingly, the mRNA and protein levels of both HDAC1 and HDAC3 gradually decreased during the EB differentiation process (Figure 2D–E), whereas the other HDACs, including HDAC2 and HDAC8, were only slightly changed (Figure 2D and 2F). Consistently, we also presented the changes in expression level of HDAC1, 2, 3, and 8 during differentiation without LIF (Figure 2G and 2H), which further confirmed that HDAC1 and HDAC3 were down-regulated during differentiation, but not for HDAC2 and HDAC8. As shown in Figure 2C, the degree of acetylated histone H4 gradually increased during EB differentiation, suggesting that the increased histone acetylation is probably due to decreased HDAC1 and HDAC3 expression in the differentiation.

### Loss of HDAC1 or 3 enhances mesodermal lineage differentiation

**Figure 3:**
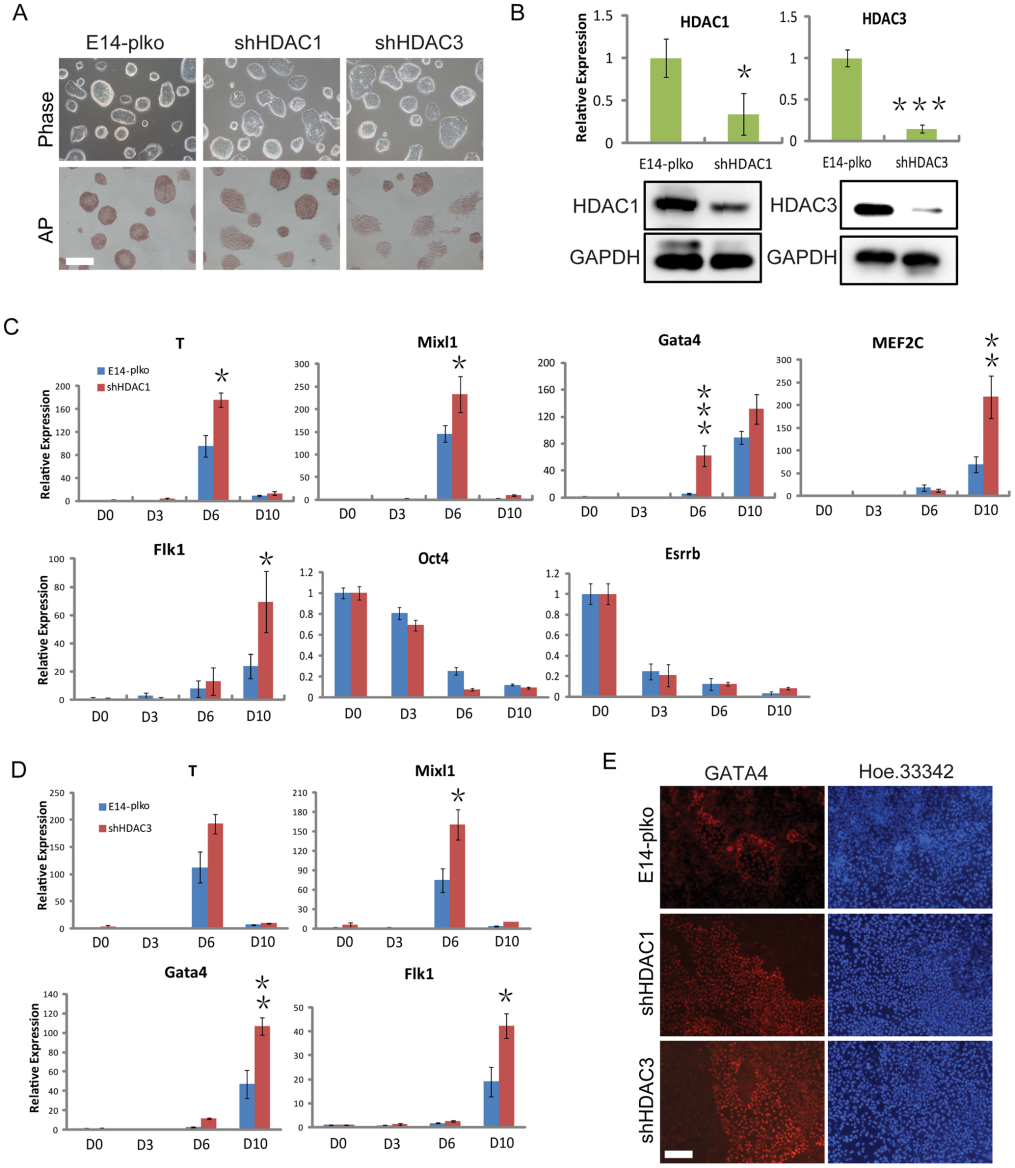
Loss of HDAC1 or 3 enhances mesodermal lineage differentiation. (*A*) Bright-field images and alkaline phosphatase staining of ESCs in shHDAC1 and shHDAC3 ESCs. (*B*) Western blotting verification and QRT-PCR analysis of the knockdown of HDAC1 and HDAC3 in stable E14 cell lines. GAPDH was used as a loading control. (*C*) QRT-PCR analysis of mesoderm genes in shHDAC1 ESCs and control cells at the days 0, 3, 6, and 10 during EB differentiation. (*D*) QRT-PCR analysis of mesoderm genes in shHDAC3 ESCs and control cells during EB differentiation. (*E*) Representative immunofluorescence images for the GATA4 expression level in control, shHDAC1, and shHDAC3 cells after 9 days of EB formation. Green, Gata4; blue, Hoechst 33342 for nuclei staining. Data are expressed as means ± SD. Statistical significance was assessed by two-tailed Student's t test. ***, P<0.001; **, P<0.01; *, P<0.05.

To elucidate further whether the changes in HDAC1 and HDAC3 expression were associated with EB differentiation, we first established the stable cell lines of HDAC1 and 3 knockdown using shRNAs (Figure 3A). The expression levels of HDAC1 and 3 were efficiently reduced by 75% and 85%, respectively (Figure 3B). We observed marked changes in the morphology of HDAC1 and 3 knockdown ESCs (shHDAC1 and shHDAC3, respectively) when we passaged these stable cell lines. Both the shHDAC1 and shHDAC3 cell lines exhibited more significant flattened state than the control cells (Figure 3A). It could also be found that HDAC1 and 3 were important for stem cells to maintain the undifferentiated phenotype in AP staining. To identify the cell types present in control and shHDAC1 cells during EB differentiation, we collected RNA at time points from day 0 to 10 and then performed QRT-PCR to detect pluripotency markers and lineage-specific markers. The pluripotency markers Oct4 and Esrrb were repressed in both control and shHDAC1 cells (Figure 3C). The key regulators of mesodermal specification, such as T, Mixl1, Gata4, and Flk1, were enhanced in EBs lacking HDAC1 compared with those in control EBs. Consistent with the increased level of mesodermal gene, we also monitored the increased expression of the cardiomyocyte-specific marker Mef2c (Figure 3C). The ectoderm lineage markers were slightly enhanced by knockdown of HDAC1, while there were no significant changes in endoderm genes (Figure S1A). Similarly, the marker genes for the mesoderm lineage, including T, Mixl1, Gata4, and Flk1, were enhanced in EBs lacking HDAC3 compared with those in control EBs (Figure 3D). The expression of other two lineage markers was also detected in EBs lacking HDAC3, and those data might show that shHDAC3 could affect the expression of endoderm genes, but no ectoderm (Figure S1B). We also demonstrated that both the shHDAC1 and shHDAC3 cells increased the Gata4 expression in the EB differentiation compared with that in control EBs by immunostaining (day 10) (Figure 3E). Since the continuous expression of HDAC2 and 8 might also be involved in the differentiation, we tried to investigate whether knockdown of HDAC2 and 8 would potentiate EB differentiation. Our data indicated that knockdown of HDAC2 or 8 in ES cells did not affect the expression of three lineage markers significantly (Figure S2A–B). Thus, our findings demonstrated that knockdown of either HDAC1 or 3 enhanced the differentiation to the mesodermal lineage.

### Ectopic expression of HDAC1 and 3 inhibits the differentiation into the mesodermal lineage in EBs

**Figure 4:**
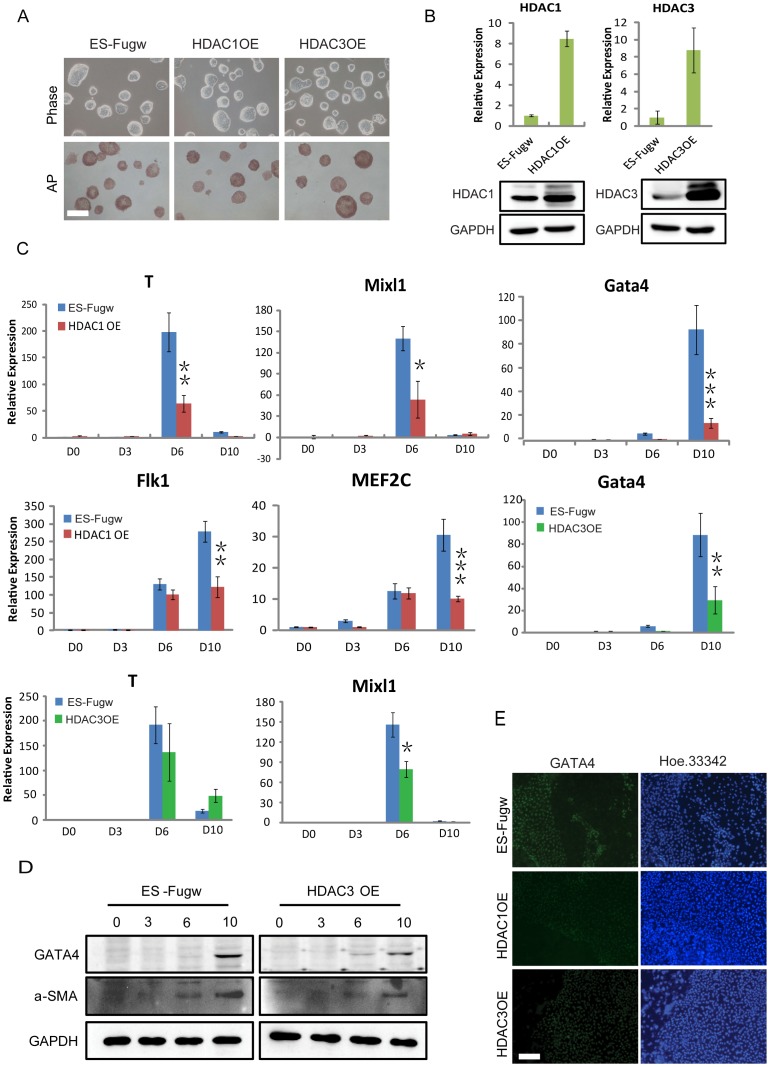
Ectopic expression of HDAC1 and 3 inhibits the differentiation into the mesodermal lineage in EBs. (*A*) Bright-field images and alkaline phosphatase staining of ESCs in control, HDAC1-overexpression (HDAC1-OE), and HDAC3-overexpression (HDAC3-OE) ESCs. (*B*) Western blotting verification and QRT-PCR analysis of the overexpression of HDAC1 and HDAC3 in stable E14 cell lines. GAPDH was used as a loading control. (*C*) QRT-PCR analysis for the mRNA levels of mesoderm genes in HDAC1-OE ESCs, HDAC3-OE ESCs and control cells during EB differentiation. (*D*) Western blotting analysis of the Gata4 and α-SMA protein levels in HDAC3-OE ESCs and control cell lines during EB differentiation. (*E*) Representative immunofluorescence images for the GATA4 expression level in control, HDAC1-OE, and HDAC3-OE cells after 9 days of EB formation. Red, Gata4; blue, Hoechst 33342 for nuclei staining. Data are expressed as means ± SD. Statistical significance was assessed by two-tailed Student's t test. ***, P<0.001; **, P<0.01; *, P<0.05.

To elucidate directly whether the changes in HDAC1 and 3 expression levels were associated with mesodermal lineage commitment, we investigated whether overexpression of HDAC1 and 3 could affect ESC differentiation to the mesodermal lineage. We could stably overexpress HDAC1 (HDAC1-OE) and HDAC3 (HDAC3-OE) in ESCs (Figure 4A). The effect of HDAC1 and HDAC3 overexpression was shown in Figure 4B. Under EB differentiation condition, both HDAC1-OE and HDAC3-OE EBs displayed reduced levels of T, Mixl1, and Gata4 compared with the control cells (Figure 4C). Consistently, HDAC3-OE cells presented lower expression level of Gata4 and a-SMA compared with that in control cells at the indicated days 0, 3, 6, and 10 of EB differentiation (Figure 4D). As expected, We further demonstrated that HDAC1-OE and HDAC3-OE ESCs differentiation showed reduced level of Gata4 protein by immunostaining (Figure 4E). These findings suggested that both HDAC1 and 3 suppressed the differentiation capacity of ESCs toward mesoderm lineage.

### The histone deacetylase activity of HDACs is indeed required for the regulation of mesoderm genes

**Figure 5:**
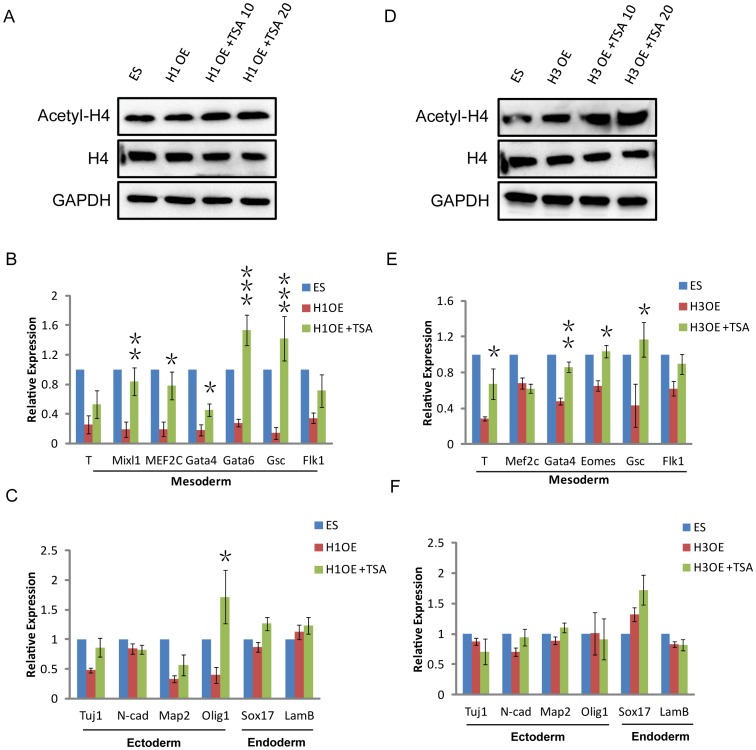
The histone deacetylase activity of HDACs is required for the regulation of mesoderm gene. (*A*) Western blotting verification of acetyl-H4 and H4 expression levels in control, HDAC1-OE (H1 OE), and TSA-treated H1-OE cells. GAPDH was used as a loading control. (*B, C*) QRT-PCR analysis of the three germ layer genes at day 6 of EB differentiation in control, H1-OE, and TSA-treated H1-OE cells. (*D*) Western blotting verification of acetyl-H4 and H4 expression levels in control, HDAC3-OE (H1 OE), and TSA-treated H3-OE cells. GAPDH was used as a loading control. (*E, F*) QRT-PCR analysis of the three germ layer genes at day 6 of EB differentiation in control, H3-OE, and TSA-treated H3-OE cells. Data are expressed as means ± SD. Statistical significance was assessed by two-tailed Student's t test. ***, P<0.001; **, P<0.01; *, P<0.05.

To confirm the function of HDACs in mesoderm differentiation, we analyzed whether histone deacetylase activity was required for its regulation of mesoderm gene expression. We used the HDAC activity inhibitor TSA to treat HDAC1-OE and HDAC3-OE cells in the process of EB differentiation. We showed that the protein level of acetyl-H4 significantly increased following TSA treatment in both HDAC1-OE and HDAC3-OE cells (Figure 5A and 5D). QRT-PCR analysis showed that TSA could rescue the effect of HDAC1 overexpression in regulating the mRNA level of mesoderm genes in EB differentiation (Figure 5B), but no marked change was noted for ectoderm and endoderm genes (Figure 5C). As expected, We found that the mRNA level of genes associated with mesoderm differentiation showed similar results in the TSA-treated HDAC3-OE EBs (Figure 5E–F), suggesting that the histone deacetylase activity of HDACs was required in this process.

### HDAC could repress the transcriptional activity of T/Bry via physical interaction

**Figure 6:**
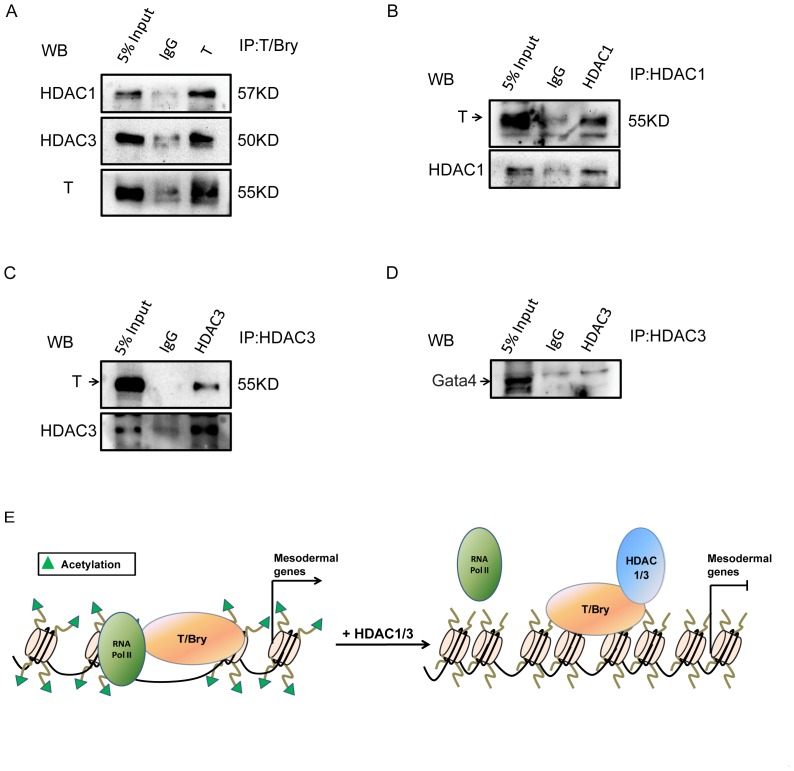
HDAC can repress the transcriptional activity of T/Bry via physical interaction. (*A*) HDAC1 and HDAC3 interact with the T-box transcription factor T/Bry. Co-immunoprecipitation (Co-IP) was performed using control IgG or T/Bry antibody, followed by western blot analysis for HDAC1 and HDAC3. 5% Input (v/v) indicated that the ratio between the loading sample and precipitation is one to twenty. (*B*) Co-IP was performed using control IgG or HDAC1 antibody, followed by western blot analysis for T/Bry. (*C*) Co-IP was performed using control IgG or HDAC3 antibody, followed by western blot analysis for T/Bry. (*D*) HDAC3 does not interact with Gata4. Co-IP was performed using control IgG or HDAC3 antibody, followed by western blot analysis for Gata4. (*E*) A summary model shows the mechanism of HDACs in regulating the expression of mesodermal genes.

HDACs had been reported to act as repressors to inhibit transcriptional factors, impairing development. Previous research had revealed that NKX2.5 directly interacted with HDAC1 to repress certain target genes, resulting in hypo-acetylation at the promoters of cardiac genes [22]. To further determine the effects of HDACs and dissect their mechanisms in regulating mesoderm differentiation, we hypothesized that unrevealed transcriptional factors might mediate the transcriptional function of HDACs in mesodermal differentiation. To confirm this hypothesis, we performed co-IP assay to determine whether endogenous HDACs could be immunoprecipitated with the candidate mesodermal transcription factors. After expressing the T/Bry (a key mesoderm marker) in the cells, we found that both HDAC1 and HDAC3 could interact with T/Bry by co-IP (Figure 6A). Consistent results were also shown in Figure 6B and 6C, in which co-IP assay was performed using HDAC1 or HDAC3 antibody. In addition, we demonstrated the lack of interaction between HDAC3 and Gata4, which was also crucial for mesoderm differentiation (Figure 6D). These results imply that T/Bry might mediate the recruitment of HDAC1 or 3 to regulate mesodermal lineage commitment. A summary model showed the mechanism of HDACs in regulating the expression of mesodermal genes (Figure 6E). Briefly, HDACs could directly interact with mesoderm lineage factor T/Bry, which caused hypo-acetylation at the promoters of T/Bry-targeted genes, then blocked mesoderm genes expression and lineage commitment.

## Discussion

Histone modifications have constituted major mechanisms of gene expression during differentiation and development [3]–[5] and have been implicated in the regulation of various biological processes, including cell cycle regulation [6], cell differentiation [25],[26], and cancer [27]. In our study, we showed that each of the class I HDAC members has its own pattern of expression during the early differentiation of ESCs. Among the class I HDACs, the expression levels of HDAC2 and 8 are mostly maintained during EB differentiation. Previous study has also shown that HDAC2 and HDAC1 are most similar (approximately 80% amino acid identity) in the class I HDACs [13]. However, HDAC2-deleted mice could survive until a short time after birth, demonstrating that HDAC2 is essential for complete mouse development, particularly for the later stage of development. The distinct mechanism of HDAC2 in mammalian development remains poorly understood. HDAC8-deficient mice could survive for some time without a severe phenotype, a result that is consistent with a stable expression pattern during EB differentiation, showed that HDAC8 might not be a determinant molecule in mammalian development. Consistent with the HDAC1 and 3 knockout phenotype of mice [14],[16], our results showed that the expression levels of both HDAC1 and 3 are decreased and that both of them function during EB differentiation. These data suggested that HDAC members could cooperatively regulate the common downstream genes because of their similar histone deacetylase activities and their individual specific mechanisms in differentiation and development. HDACs function in stem cell pluripotency and differentiation, which act on the molecular network controlling the maintenance of the pluripotent state and commitment to a lineage. In our study, loss-of-function experiments showed that loss of HDAC1 leads to the differentiation of ESCs toward mesodermal and ectodermal lineages, while loss of HDAC3 leads to the enhancement of ESC differentiation into mesoderm under these conditions. Our data indicated that class I HDACs (only HDAC1 and 3) are obviously involved in regulating mesoderm differentiation and clearly demonstrated that the functions of HDACs are diverse and isoform-dependent, particularly in the transcriptional regulation of differentiation genes.

A previous study has demonstrated that NKX2.5, which is essential for cardiomyocyte differentiation, interacts with p300 physically [28]. A direct interaction also exists between NKX2.5 and HDAC1, resulting in the repression of transcriptional activity [22]. Class II HDACs also directly bind and repress MEF2 during cardiac mesoderm differentiation [29]. The T-box transcription factor T/Bry, a classical and conserved mesodermal factor, is critical for primitive streak (PS) formation [30]. Our studies described in this report implied that T/Bry might mediate the recruitment of HDAC1 in mesodermal differentiation. During the early stage of embryonic development, the three germ layers (endoderm, mesoderm and ectoderm) are specified in gastrulation, which begins with PS formation [31],[32]. Our data showed that T/Bry might also recruit HDAC3 to repress certain target genes in mesodermal differentiation, suggesting that the HDAC3 deletion results in gastrulation-deficient mice are partially due to the interruption of interplay between T and HDAC3. Collectively, we have currently provided more information about the complexity of HDACs in modulating mesodermal differentiation.

HDAC inhibitors are small molecules that can inhibit the activities of HDACs, efficiently and temporally affect the gene expression and cause directed differentiation of embryonic and multipotent stem cells to specific cellular lineages, including the neuronal, cardiomyocytic, and hepatic lineages [33]–[38]. HDAC inhibitors have also shown remarkable therapeutic potential in various diseases, including cancer, neurological diseases [37],[38], bone diseases [39] and cardiac diseases [40]. Recent studies have suggested that the primary substrates of HDAC enzymes are not only histone but also non-histone proteins [2],[41]. Therefore, inhibitors of these HDACs are attractive targets in ESC differentiation, particularly in mesoderm differentiation. Additionally, treatment of differentiated EBs with TSA promotes cardiomyocyte differentiation by increasing Nkx2.5 expression [5]. Our data showed that TSA treatment at the early stage of differentiation significantly enhances the expression levels of mesoderm genes such as T, Gata4, and Mef2c in both monolayer and EB differentiation conditions. Although all of the class I HDAC members are inhibited by TSA, only HDAC1 and 3 are obviously involved in regulating mesoderm differentiation. Differences in the regulatory mechanisms and molecular targets exist among the HDAC members, even among the same class. A better understanding of HDAC-related events leading to lineage differentiation might clarify the pathways regulating the different cell fates and patterning of the early embryo in the future. Additionally, it is helpful to understand the knockout phenotypes of individual HDACs in mice. Furthermore, a better understanding of the function of individual HDACs in the differentiation of ESCs could encourage efforts to develop isoform-selective HDAC inhibitors with better specific HDAC inhibitory potency. Such studies might also be very useful for HDACi therapy considering the restrictions and limitations of HDACi clinical application because of the adverse side effects of HDACi due to the lack of isoform specificity.

## Materials and Methods

### Cell Culture

ESCs (E14T) were cultured on 0.1% gelatin-coated plates in ESC medium consisting of DMEM (Gibco) containing 15% (v/v) fetal bovine serum (FBS; Gibco), 2 mM L-glutamine (Hyclone), 100 µM nonessential amino acids (NEAAs) (Hyclone), 0.1 µM 2-mercaptoethanol (Gibco), 1 mM sodium pyruvate and leukemia inhibitory factor (LIF; 1000 U/ml; Chemicon). The ESCs were fed with fresh medium every day and passaged every 2–3 days using 0.25% trypsin/EDTA (Gibco). The 293FT cells were maintained in DMEM supplemented with 10% (v/v) FBS.

### Vectors and Viral Infection

The HDAC1 and HDAC3 overexpression plasmids (Fuw-HDAC1 and Fuw-HDAC3, respectively) were generated by cloning the HDAC1 or HDAC3 coding region into the Fuw vector, in which HDAC1 or HDAC3 coding sequence was derived by the Ubiquitin promoter. To construct shHDAC1 or shHDAC3, 21-base pair HDAC1- or HDAC3-specific regions (shHDAC1, AAGCAGCGTCTCTTTGAGAAC; shHDAC3, AACCTCATCGCCTGGCATTGA) for RNA interference were designed and cloned into the pLKO.1 cloning vector derived by U6 promoter. All the vectors were purchased from Addgene and the constructed plasmids were verified by DNA sequencing. All the primers used in this study are listed in Table S1.

To generate the lentivirus, 293FT cells were seeded at a density of 1.2×10^5^ cells per well in 6-well plates. Lentiviral vectors were introduced into 293FT cells using the Fugene HD transfection reagent (Roche) according to the manufacturer's recommendations. Foreign DNA (1.5 µg) was transfected into 293FT cells together with the packaging plasmids PAX2 (1.125 µg) and VSV-G (0.75 µg). The virus-containing medium was harvested at 48 h after transfection. To establish the HDAC1 or HDAC3 knockdown cell line, ESCs were infected with shHDAC1 or shHDAC3 lentivirus respectively, and then positive cells were selected using puromycin (1 µg/ml). To establish the HDAC1 or HDAC3 overexpression cell line, we infected ESCs with Fuw-HDAC1 and Fuw-HDAC3 lentivirus. The monoclonal ESC lines were chosen manually.

### RNA Extraction and Quantitative RT-PCR (QRT-PCR)

Total RNA was extracted using RNAiso plus (Takara Bio Inc, Japan) and was subsequently used to synthesize cDNA using the PrimeScript RT reagent kit (Takara). QRT-PCR analysis was performed using the SYBR Green qPCR Master Mix (Takara). For QRT-PCR, a template equivalent to 20 ng of total RNA was subjected to 40 cycles of quantitative PCR, and the expression levels of the genes of interest were normalized to that of the *gapdh* gene. The relative expression level was calculated by the 2^−_△△_^Ct^^ method [42].

### ESCs Differentiation

In –LIF differentiation assay, ESCs were replated on the gelatin-coated 6-well plate in ESC medium (8×10^4^ cells/well) for 24 h. After 24 h, the medium was replaced by ESC medium without LIF. The samples were collected at the indicated days 0, 2, 3, and 4 of differentiation.

In the EB formation differentiation assay, ESCs were harvested by trypsinization (0.25% trypsin/EDTA), and aliquots of 2×10^5^ cells were resuspended in 60-mm bacterial culture dishes in ESC medium without LIF to generate EBs. The medium was changed every day, and a portion of the cells was discarded to maintain the proper density. For immunostaining, the generated EBs were seeded onto 24-well plates at day 4 of differentiation and cultured on the gelatin-coated 24-well plates for another 6 days. RNA was extracted from the EBs at the indicated days of differentiation and used for QRT-PCR analyses. The primers used for QRT-PCR are listed in Table S2.

### Western Blotting

The cells were washed twice with ice-cold phosphate-buffered saline (PBS) and incubated on ice for 30 min with SDS lysis buffer. Equal amounts of cell lysates were separated by SDS-PAGE. Primary antibodies, including anti-Oct4 (Santa Cruz Biotechnology), anti-Nanog (Abcam), anti-HDAC1 (Sigma), anti-HDAC3 (Cell Signaling), anti-HDAC2 (Santa Cruz Biotechnology), anti-HDAC8 (Santa Cruz Biotechnology), anti-H4/acetyl-H4 (Millipore) anti-H3/acetyl-H3 (Millipore), anti-T/Bry (abcam), anti-Gata4 (Santa Cruz Biotechnology), anti-SMA (Sigma) and anti-GAPDH (Sigma) were used in this study. GAPDH was used as loading controls. After incubation with the appropriate secondary antibodies, signals were visualized by enhanced chemiluminescence (ECL) (ImageQuant LAS 4000 mini).

### Alkaline Phosphatase Staining and Immunostaining

AP staining was carried out using the FastRed Alkaline Phosphatase Kit (Sigma) according to the manufacturer's protocol.

In the immunostaining assay, the cells were washed with PBS, fixed with 4% paraformaldehyde (4% PFA) at room temperature for 20 min and then permeabilized with 0.2% Triton X-100 for 8 min. The cells were blocked for 1 h in 10% FBS (v/v in PBS), incubated overnight with primary antibodies (anti-Gata4; Santa Cruz Biotechnology) at 4°C, and then washed three times with 10% FBS (v/v in PBS). Next, the cells were stained with fluorescent secondary antibodies in the dark for 1 h and counterstained with Hoechst 33342 for 10 min at room temperature. Finally, the cells were examined under a fluorescence microscope to acquire fluorescent images.

### Co-immunoprecipitation (Co-IP)

The ESCs were collected and lysed with lysis buffer (1% Triton X-100 in 50 mM Tris-HCl (pH 7.4) containing 150 mM NaCl, 2 mM Na3VO_4_, 100 mM NaF, and protease inhibitors). Next, the cell lysates were incubated with anti-HDAC1 or HDAC3-coated A/G beads (Sigma) for 4 h or overnight. The beads were washed three times with lysis buffer, protein complexes were eluted by boiling in SDS loading buffer (250 mM Tris-HCl, pH 6.8; 10% (w/v) SDS; 0.5% (w/v) Bromophenol Blue; 50% (v/v) Glycerin; 5% (v/v) β-ME), and the immunoprecipitates were analyzed by western blotting with antibodies as specified.

### Statistical Analysis

The error bars represent the standard deviation (SD) of three independent experiments. *, **, and *** indicate P<0.05, P<0.01, and P<0.001, respectively (two-tailed Student's t test).

## Supporting Information

Figure S1 **Ectoderm and endoderm lineage markers analysis of HDAC1 and 3 knockdown during EB differentiation.** (*A*) QRT-PCR analysis of ectoderm and endoderm lineage markers in shHDAC1 ESCs and control cells during EB differentiation. (*B*) QRT-PCR analysis of ectoderm and endoderm markers in shHDAC3 ESCs and control cells during EB differentiation.
(TIF)

Figure S2 **Lineage markers analysis of HDAC2 and 8 knockdown during EB differentiation.** (*A*) QRT-PCR analysis of lineage markers in shHDAC2 ESCs and control cells during EB differentiation. (*B*) QRT-PCR analysis of lineage markers in shHDAC8 ESCs and control cells during EB differentiation.
(TIF)

Table S1 **Primers used for vectors construction.**
(DOC)

Table S2 **Primer sets used in qRT-PCR assays.**
(DOC)
