# Supplementary figures and images for: Histone Deacetylase 1 and 3 Regulate the Mesodermal Lineage Commitment of Mouse Embryonic Stem Cells
Source: PLoS One. 2014 Nov 20;9(11):e113262. doi: 10.1371/journal.pone.0113262 (PMC4239075; doi:10.1371/journal.pone.0113262)

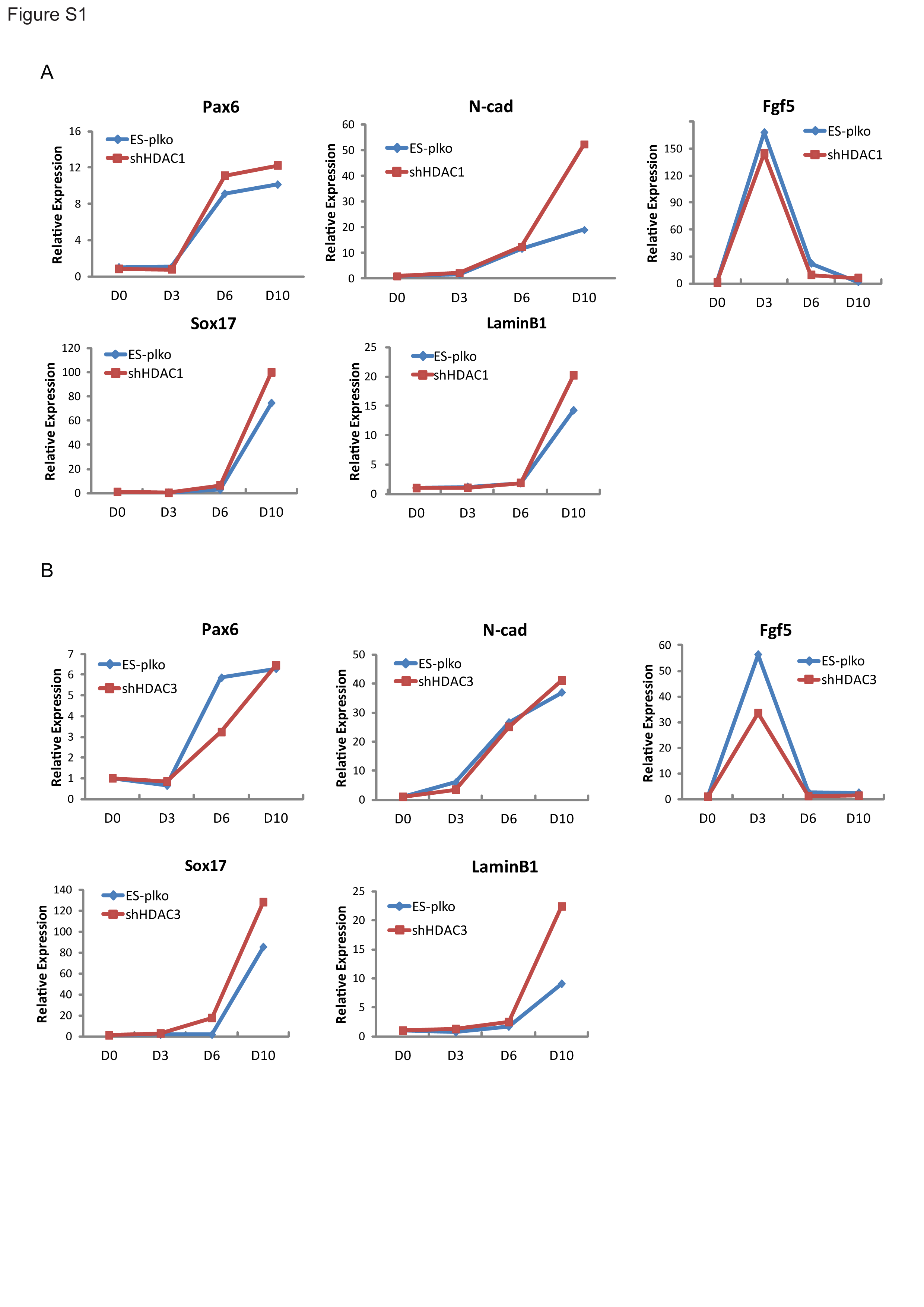

Supplement: Figure S1 — Ectoderm and endoderm lineage markers analysis of HDAC1 and 3 knockdown during EB differentiation. (A) QRT-PCR analysis of ectoderm and endoderm lineage markers in shHDAC1 ESCs and control cells during EB differentiation. (B) QRT-PCR analysis of ectoderm and endoderm markers in shHDAC3 ESCs and control cells during EB differentiation. (TIF) [file pone.0113262.s001.tif]

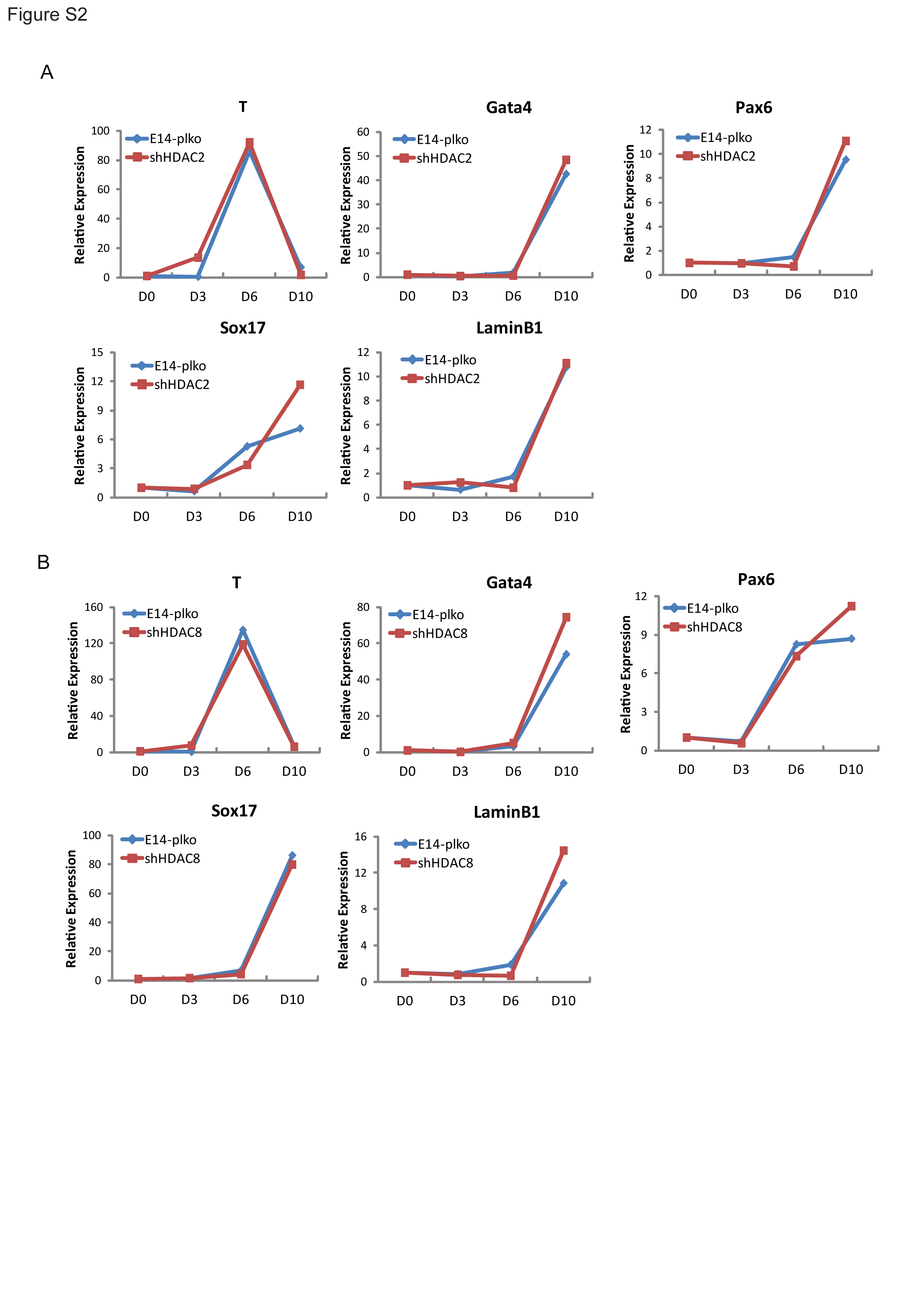

Supplement: Figure S2 — Lineage markers analysis of HDAC2 and 8 knockdown during EB differentiation. (A) QRT-PCR analysis of lineage markers in shHDAC2 ESCs and control cells during EB differentiation. (B) QRT-PCR analysis of lineage markers in shHDAC8 ESCs and control cells during EB differentiation. (TIF) [file pone.0113262.s002.tif]
