# Supplementary material for: Histone Deacetylase 1 and 3 Regulate the Mesodermal Lineage Commitment of Mouse Embryonic Stem Cells
Source: PLoS One. 2014 Nov 20;9(11):e113262. doi: 10.1371/journal.pone.0113262 (PMC4239075; doi:10.1371/journal.pone.0113262)
Supplement: Table S1 — Primers used for vectors construction. (DOC) [file pone.0113262.s003.doc]

**Table S1. Primers used for vectors construction.**

| HDAC1 | CACAGGGATCCATGGCGCAGACTCAGG | F |
| --- | --- | --- |
|  | CACAGGAATTCTCAGGCCAACTTGACCTC | R |
| HDAC3 | CACAGACCGGTATGGCCAAGACCGTGGCG | F |
|  | CACAGGAATTCCTAAATCTCCACATCACTTTC | R |
| shHDAC1 | AAGCAGCGTCTCTTTGAGAAC |  |
| shHDAC3 | AACCTCATCGCCTGGCATTGA |  |
| T | CACAG ACCGGT ATGAGCTCGCCGGGCACAG | F |
|  | CACAG GAATTC TCACATAGATGGGGGTGACACAG | R |
| shHDAC2 | GTATCATCAGAGAGTCTTATT |  |
| shHDAC8 | GGGAATATTACGATTGCGACG |  |
