# Supplementary material for: Histone Deacetylase 1 and 3 Regulate the Mesodermal Lineage Commitment of Mouse Embryonic Stem Cells
Source: PLoS One. 2014 Nov 20;9(11):e113262. doi: 10.1371/journal.pone.0113262 (PMC4239075; doi:10.1371/journal.pone.0113262)
Supplement: Table S2 — Primer sets used in qRT-PCR assays. (DOC) [file pone.0113262.s004.doc]

**Table S2.** Primer sets used in qRT-PCR assays.

| HDAC1 | GGGCACCAAGAGGAAAGTCT | F |
| --- | --- | --- |
|  | AGCAAATTGTGAGTCATGCG | R |
| HDAC2 | CATGGCGTACAGTCAAGGAG | F |
|  | TCATCCGGATTCTATGAGGC | R |
| HDAC3 | AGTCAGCCCCACCAATATGC | F |
|  | CCTGTGTAACGGGAGCAGAACT | R |
| HDAC8 | TTTGAGCGTATTCTCTACGTGGA | F |
|  | ACACTGTAGTACCGTCCCTTC | R |
| Nanog | AGGGTCTGCTACTGAGATGCTCTG | F |
|  | CAACCACTGGTTTTTCTGCCACCG | R |
| Oct4 | TCTTTCCACCAGGCCCCCGGCTC | F |
|  | TGCGGGCGGACATGGGGAGATCC | R |
| Rex1 | GGAAGAAATGCTGAAGGTGGAGAC | F |
|  | AGTCCCCATCCCCTTCAATAGC | R |
| T | GGTGGCTTGTTCCTGGTGC | F |
|  | GTAGGTGGGCTGGCGTTAT | R |
| Gata4 | GATGGGACGGGACACTACCTG | F |
|  | ACCTGCTGGCGTCTTAGATTT | R |
| Flk1 | TTTGGCAAATACAACCCTTCAGA | F |
|  | GCAGAAGATACTGTCACCACC | R |
| Mef2c | AGCAAGAATACGATGCCATC | F |
|  | GAAGGGGTGGTGGTACGGTC | R |
| Gsc | ACCATCTTCACCGATGAGCAGC | F |
|  | CTTGGCTCGGCGGTTCTTAAAC | R |
| Pax6 | TCTTTGCTTGGGAAATCCG | F |
|  | CTGCCCGTTCAACATCCTTAG | R |
| Tuj1 | TAGACCCCAGCGGCAACTAT | F |
|  | GTTCCAGGTTCCAAGTCCACC | R |
| Neurod1 | GCTGCTGAGTCTCGGGATAG | F |
|  | GCTTGGCCTCTCTCTTTCCT | R |
| Olig1 | TGAATCCCACCTGTTTAGAGCC | F |
|  | CGATGCTCACGGATACGAGAATAG | R |
| Map2 | GGTCACAGGGCACCTATTCA | F |
|  | TGTTCACCTTTCAGGACTGC | R |
| Mash1 | AACGAGCGCGAGCGCAACCG | F |
|  | TTGGAGTAGTTGGGGGAGATG | R |
| N-cadherin | TCCTGATATATGCCCAAGACAA | F |
|  | TGACCCAGTCTCTCTTCTGC | R |
| Sox17 | CGAGCCAAAGCGGAGTCTC | F |
|  | TGCCAAGGTCAACGCCTTC | R |
| Lamin B1 | CCCCAATCTCTGTGAACCATG | F |
|  | GCAATTTGCACCGACACTGA | R |
| Mixl1 | TCCAGGATCCAGGTGTGGTT | F |
|  | GCAGGGCAATGGAGGAAAAC | R |
| Esrrb | GTCCCTCTCCGCGTTAGC | F |
|  | GGGGCAGGTTCGTCATTT | R |
| Fgf5 | AAAGTCAATGGCTCCCACGAA | F |
|  | GGCACTTGCATGGAGTTTTCC | R |
| Gata6 | GAGCTGGTGCTACCAAGAGG | F |
|  | TGCAAAAGCCCATCTCTTCT | R |
| Eomes | CCTGGTGGTGTTTTGTTGTG | F |
|  | TTTAATAGCACCGGGCACTC | R |
| GAPDH | AGGTCGGTGTGAACGGATTTG | F |
|  | TGTAGACCATGTAGTTGAGGTCA | R |
